# SHP2 mediates STAT3/STAT6 signaling pathway in TAM to inhibit proliferation and metastasis of lung adenocarcinoma

**DOI:** 10.18632/aging.205799

**Published:** 2024-05-14

**Authors:** Guojing Chai, Yingbo Nan, Haili Zhao, Qingchuan Hu

**Affiliations:** 1 Clinical Laboratory, Hebei General Hospital, Shijiazhuang 050051, Hebei, China; 2 Hebei Clinical Research Center for Laboratory Medicine, Shijiazhuang 050051, Hebei, China; 3 Hebei Key Laboratory of Molecular Medicine, Shijiazhuang 050051, Hebei, China

**Keywords:** lung adenocarcinoma, SHP2, TAM, STAT3/STAT6 signaling pathway

## Abstract

Objective: This study examines SHP2’s influence on the STAT3/STAT6 pathway in tumor-associated macrophages (TAMs) and its impact on lung adenocarcinoma proliferation and metastasis.

Methods: Lung cancer A549 and NCI-H1688 cell lines were subcutaneously injected into nude mice. Macrophages were isolated using flow cytometry and analyzed for CD163, CD206, and Arginase-1 levels via western blot. Similarly, the effect on THP1 cell-associated proteins was assessed. The impact on A549 and NCI-H1688 cell migration, invasion, and proliferation was evaluated through wound healing, Transwell assays, and CCK8.

Results: Compared to controls, the sh-RNA SHP2 group showed increased tumor volume and higher expression levels of CD163, CD206, Arginase-1, p-STAT3, p-STAT6, IL-4, IL-10, and various cathepsins in macrophages and THP1 cells. However, p-STAT1 and p-STAT5 levels remained unchanged. The sh-RNA SHP2 group also demonstrated enhanced migration, invasion, and proliferation in both cell lines.

Conclusions: SHP2 negatively affects the STAT3/STAT6 pathway in TAMs, promoting M2 polarization and cathepsin secretion, which enhances lung adenocarcinoma cell proliferation and metastasis.

## INTRODUCTION

Lung cancer stands as the predominant cause of death from cancer [1]. It is categorized into two main types based on histological differences: small cell lung cancer (SCLC) and non-small cell lung cancer (NSCLC), with the latter comprising 85% of all lung cancer cases, while SCLC accounts for 15% [2]. NSCLC itself is broken down into squamous cell carcinoma, adenocarcinoma, and large cell carcinoma [3], with squamous cell carcinomas forming 25-30% of lung cancer incidences, originating from the squamous cells in the central lung bronchi. Adenocarcinoma, the most frequent lung cancer type, represents about 40% of cases [4]. Despite advancements in understanding and treating lung adenocarcinoma, it remains highly aggressive, with a median survival time under five years [5, 6].

Tumor-associated macrophages (TAMs) play dual roles in cancer, depending on their polarization state. M1 TAMs exhibit anti-tumor effects through inflammatory mediators and cytotoxic actions, also aiding in the activation of the immune system’s response to cancer. In contrast, M2 TAMs support tumor growth and spread by enhancing angiogenesis, suppressing immune reactions, and creating a conducive environment for tumor expansion. In lung cancer, a high concentration of M2 TAMs has been linked to increased tumor aggressiveness, invasion, metastasis, and unfavorable outcomes.

The protein tyrosine phosphatase SHP2, encoded by the PTPN11 gene [7, 8], participates in several cellular functions, including gene transcription, cytokine signaling, cell differentiation, and the proliferation and migration of cancer cells. The STAT family, encompassing seven members, are crucial transcription factors activated by cytokines and growth factors, essential for various cellular processes. Of these, STAT3 is notably implicated in cancer pathogenesis and is a key player in macrophage M2 polarization, alongside STAT6 [8, 9]. It has been demonstrated that GM-CSF from cancer cells triggers STAT3 activation in TAMs, while SHP2 is capable of counteracting STAT3's activity [9, 10].

Research indicates that SHP2 negatively affects lung adenocarcinoma growth [11, 12], though the intricacies of this interaction require further exploration. This study proposes that SHP2 may dampen lung adenocarcinoma progression by inhibiting the STAT3/STAT6 pathway in TAMs, consequently reducing M2 polarization and the secretion of factors like cathepsin, which in turn, could limit the growth and spread of lung adenocarcinoma cells.

## RESULTS

### SHP2 inhibition in TAMs augments lung adenocarcinoma progression

**Figure 1:**
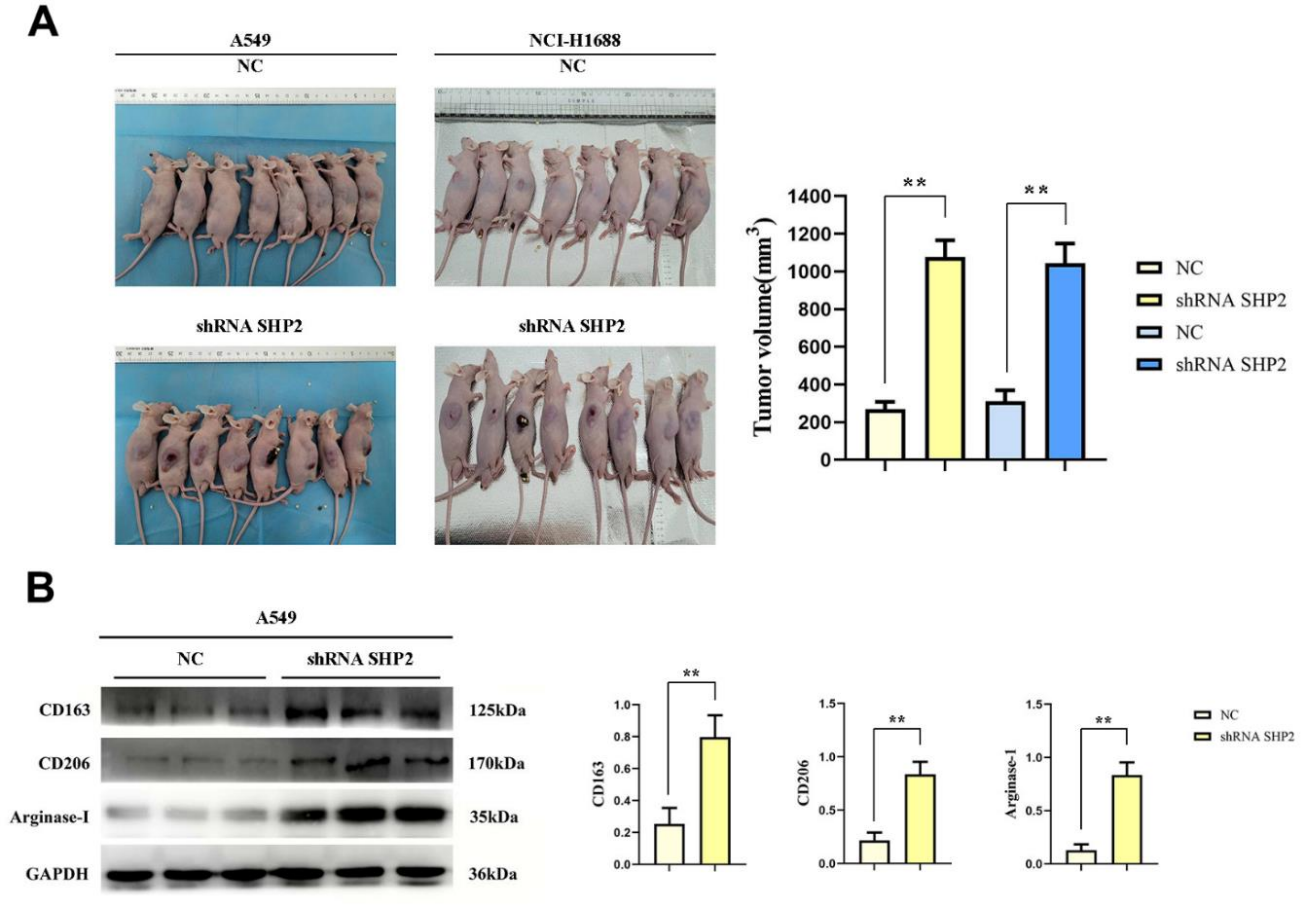
**Inhibition of SHP2 in TAM can promote M2 polarization and the development of lung adenocarcinoma.** (**A**) Subcutaneous tumor assay in nude mice and data statistics; (**B**) Western blot detects M2 markers: the expression of CD163, CD206 and Arginase-1. N=8;**P<0.01.

We examined SHP2’s role in tumor-associated macrophages (TAM) on lung adenocarcinoma by co-injecting A549 and NCI-H1688 cells with lentivirus-infected THP1 cells (comprising both control and SHP2-silenced groups) into nude mice. Tumor volume measurements revealed significantly larger tumors in the SHP2-silenced group, underscoring SHP2’s suppressive effect on lung adenocarcinoma growth (Figure 1A).

### SHP2 blockade enhances M2 macrophage polarization

Further analysis of SHP2’s influence on TAMs showed that silencing SHP2 led to increased expression of M2 macrophage markers CD163, CD206, and Arginase-1. This increase suggests SHP2 inhibition favors M2 macrophage polarization, linked to tumor-promoting conditions (Figure 1B).

### Inhibition of SHP2 can promote activation of the STAT3/STAT6 signaling pathway and secretion of cathepsin

**Figure 2:**
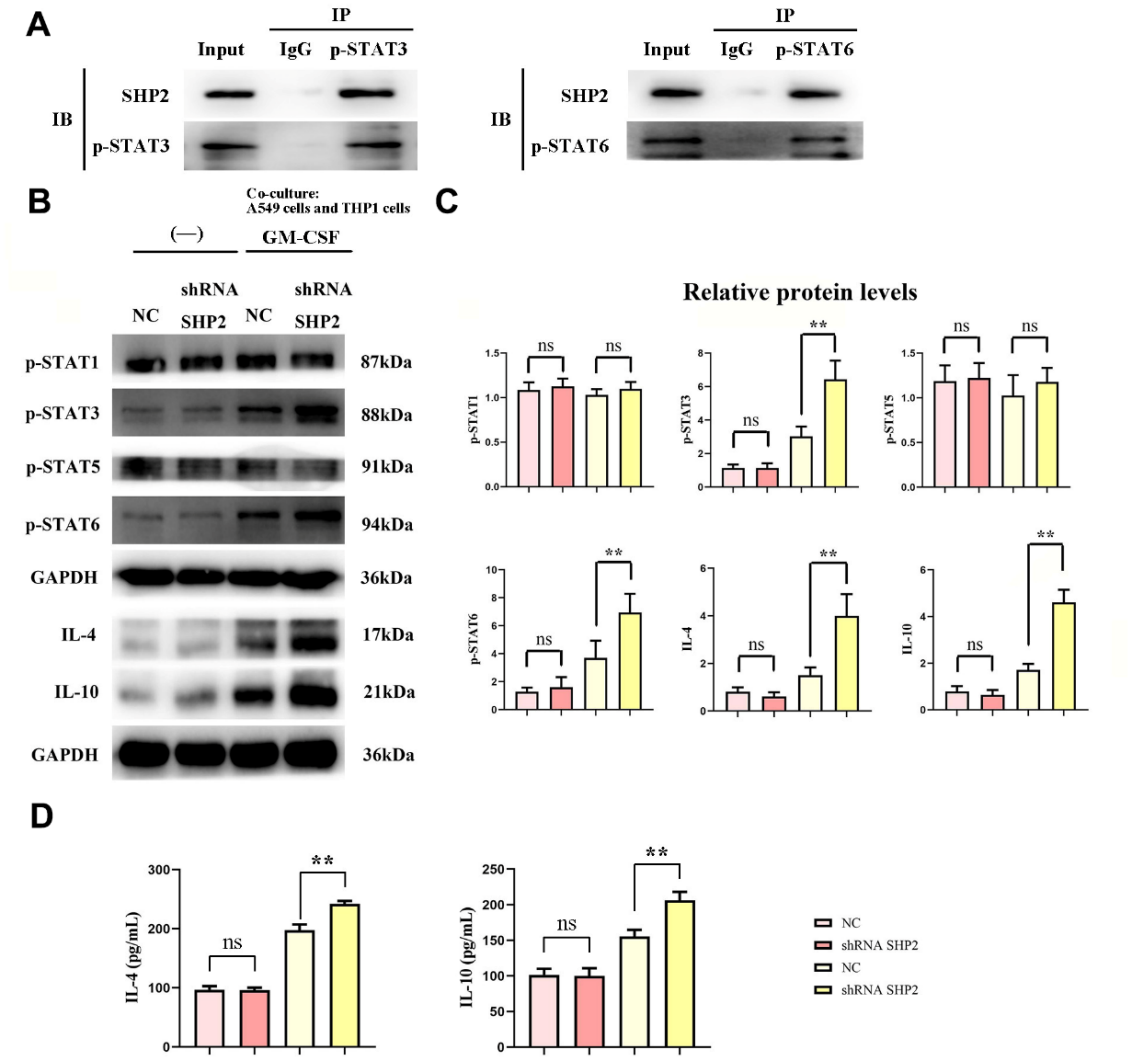
**Inhibition of SHP2 in TAM can promote the stimulation of STAT3/STAT6 signaling pathway in TAM by lung adenocarcinoma.** (**A**) Co-IP experimental results; (**B**) Protein band diagram of p-STAT1, p-STAT3, p-STAT5, p-STAT6, IL-4, IL-10; (**C**) Relative protein expression statistics of p-STAT1, p-STAT3, p-STAT5, p-STAT6, IL-4, IL-10; (**D**) ELISA detects the concentrations of IL-4 and IL- 10. N=3;**P<0.01;nsP>0.05.

**Figure 3:**
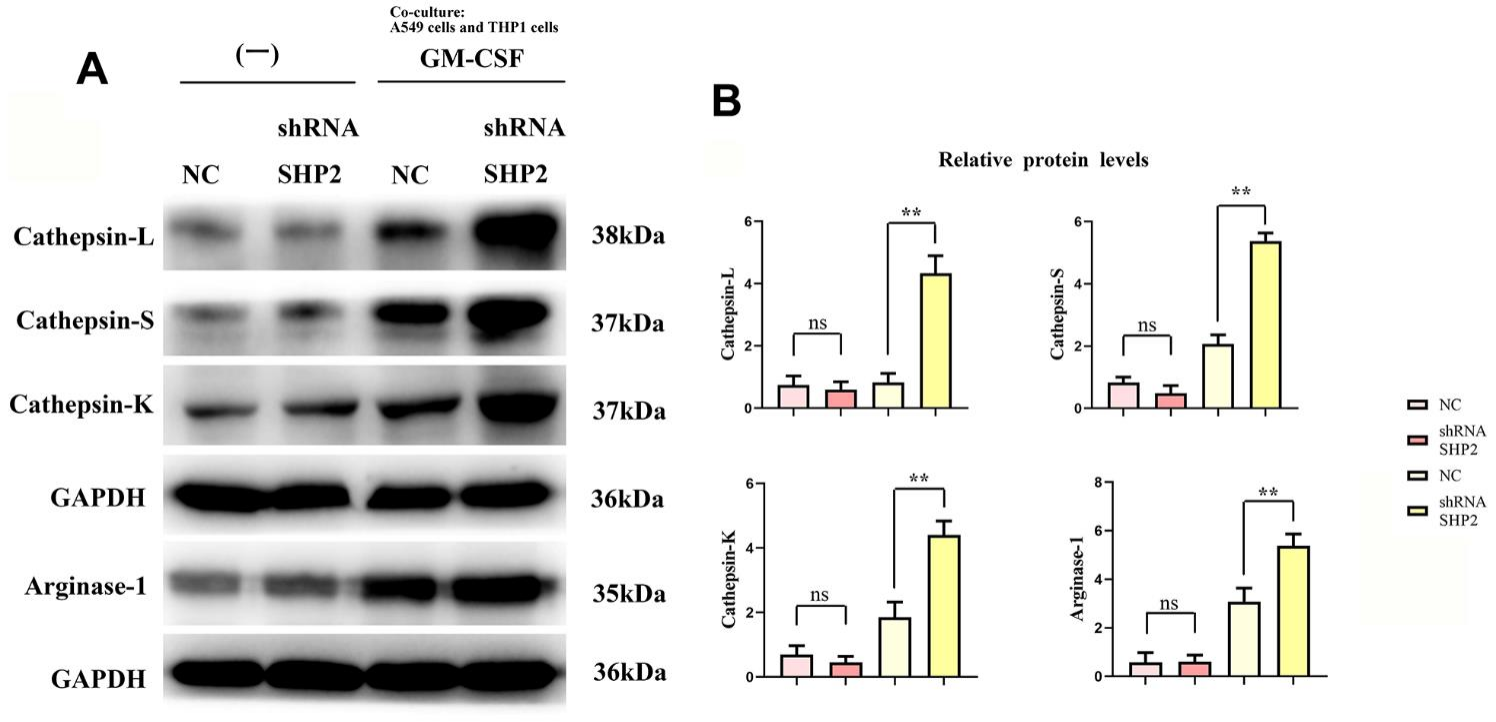
**Inhibition of SHP2 in TAM can promote the stimulation of cathepsin and M2 polarization by lung adenocarcinoma.** (**A**) Protein band diagram of Cathepsin-L, Cathepsin-S, Cathepsin-K and Arginase-1; (**B**) Relative protein expression statistics of Cathepsin-L, Cathepsin-S, Cathepsin-K and Arginase-1. N=3; **P<0.01;nsP>0.05.

We assess the effect of lung adenocarcinoma cells on TAM through detecting the expression of SHP2, p- STAT1, p-STAT3, p-STAT5, p-STAT6, IL-4, IL-10, Cathepsin-L, Cathepsin-S, Cathepsin-K and Arginase-1 in each group of THP1 cells cocultured with and without A549 cells by western blot. First, we found that the relative protein expression of SHP2 in the shRNA SHP2 group was significantly lower than that in the NC group, and the relative protein expression of SHP2 in the NC group and the shRNA SHP2 group did not change after GM-CSF stimulation of macrophages. The expression levels of p-STAT1, p-STAT3, p-STAT5, p-STAT6, IL-4, IL-10, Cathepsin-L, Cathepsin-S, Cathepsin-K and Arginase-1 in the NC group and sh-RNA SHP2 group in THP1 cells that are not cocultured with A549 cells are low and have no significant difference. The expression levels of p-STAT3, p-STAT6, IL-4, IL-10, Cathepsin-L, Cathepsin-S, Cathepsin-K and Arginase-1 in the sh-RNA SHP2 group in THP1 cells cocultured with A549 cells are significantly higher than those in the NC group. The expressions of p-STAT1 and p-STAT5 are not significantly different from those of the NC group. Co-immunoprecipitation results showed that SHP2 could bind specifically to p-STAT3 and p-STAT6. The ELISA results showed that the concentrations of IL-4 and IL-10 in THP-1 cells not co-cultured with A549 cells were lower and there was no significant difference between the NC group and the shRNA SHP2 group. In THP-1 cells co-cultured with A549 cells, the concentrations of IL-4 and IL-10 in the shRNA SHP2 group were significantly higher relative to the NC group. This indicates that GM-CSF secretion from lung adenocarcinoma cells can stimulate the activation of STAT3/STAT6 signaling pathway in TAM and promote the secretion of cathepsin, which is exacerbated by the inhibition of SHP2 (Figures 2, 3).

### Inhibition of SHP2 in TAM can enhance the migration and invasion of lung adenocarcinoma cells

**Figure 4:**
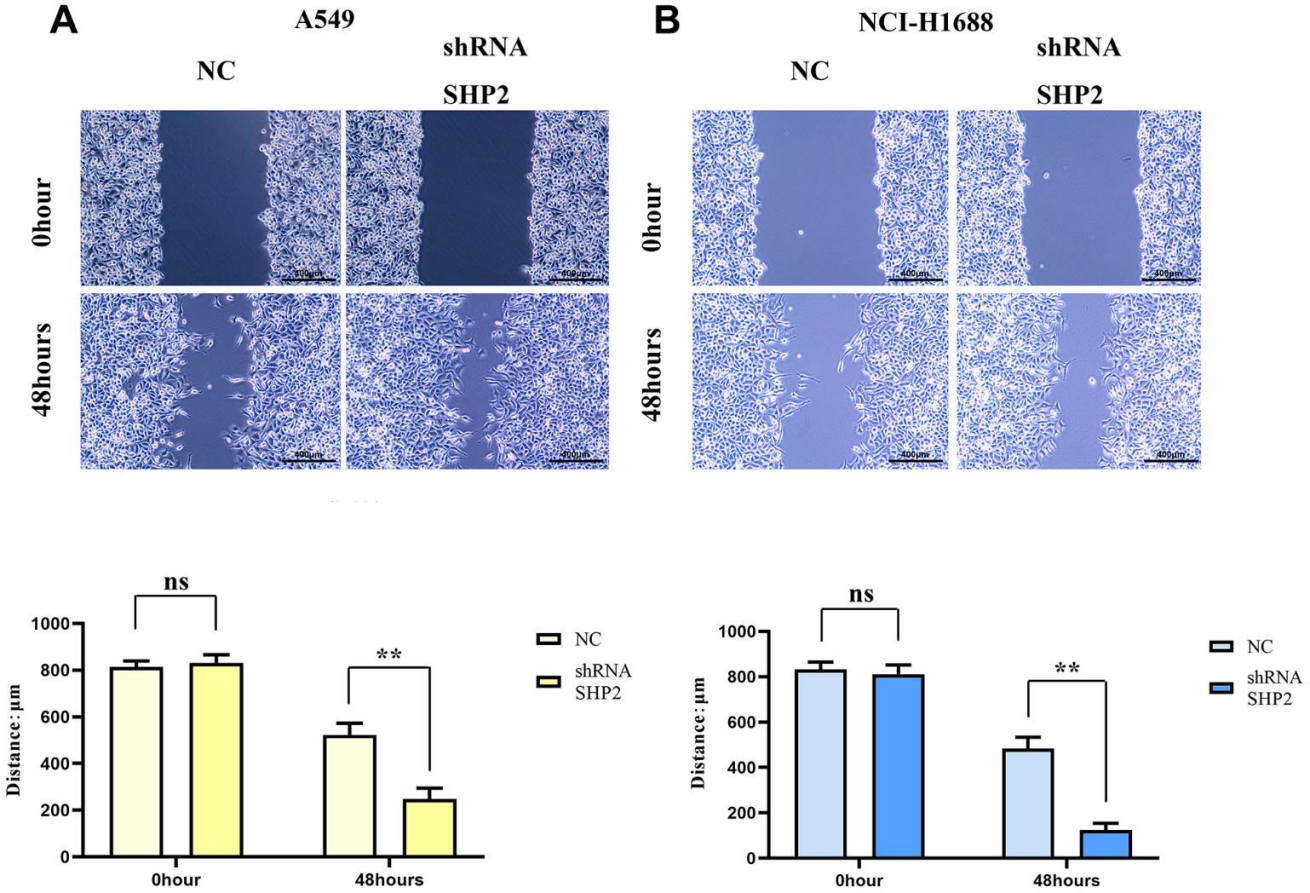
**Inhibition of SHP2 in TAM can enhance the migration ability of lung adenocarcinoma cells.** (**A**) Cells healing assay and data statistics of A549 cells; (**B**) Cells healing assay and data statistics of NCI-H1688 cells. 40×,1824 pixels×1216 pixels,400 μm;N=3;**P<0.01;nsP>0.05.

**Figure 5:**
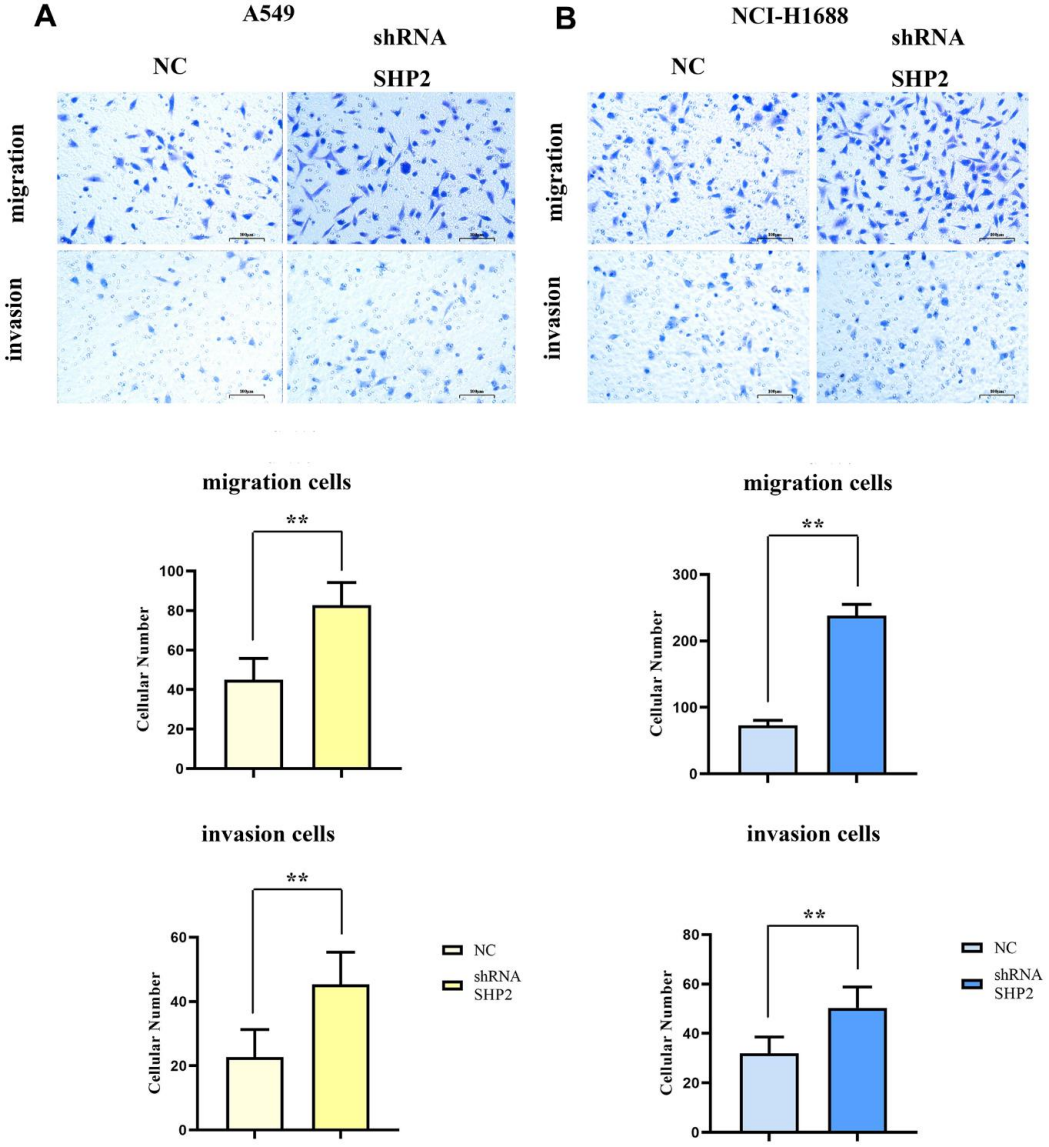
**Inhibition of SHP2 in TAM can enhance the migration and invasion ability of lung adenocarcinoma cells.** (**A**) Transwell diagram and data statistics of A549 cells; (**B**) Transwell diagram and data statistics of NCI-H1688 cells. 100×,1824 pixels×1216 pixels,100 μm;N=3;**P<0.01

To evaluate the effects of SHP2 in TAM on the migration and invasion abilities of lung adenocarcinoma cells, we coculture transfected THP1 cells with A549 cells and NCI-H1688 cells respectively, and then detect the migration and invasion abilities of A549 and NCI-H1688 cells using cell healing assay and Transwell. The results show that the cell scratch spacing in the sh-RNA SHP2 group is significantly reduced in the 48th hour relative to the NC group, indicating that the inhibition of SHP2 in TAM can enhance the migration ability of lung adenocarcinoma cells. Moreover, the number of the migration and invasion cells in the sh-RNA SHP2 group is significantly higher than that in the NC group, which indicates that the inhibition of SHP2 in TAM can enhance the migration ability and invasion capacity (Figures 4, 5).

### Effect of SHP2 inhibition on lung adenocarcinoma cell proliferation

**Figure 6:**
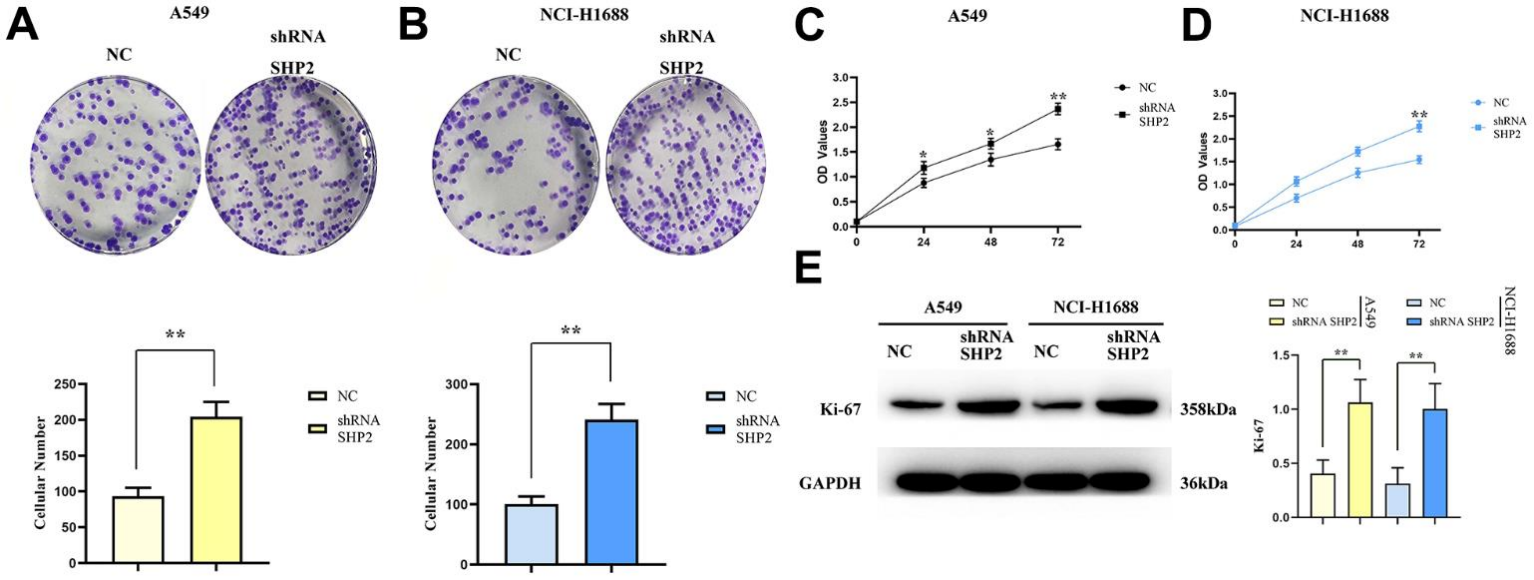
**Inhibition of SHP2 in TAM can enhance the proliferation ability of lung adenocarcinoma cells.** (**A**) Monoclonal proliferation experiment results and data statistics of A549 cells; (**B**) CCK8 test results of A549 cells; (**C**) Monoclonal proliferation experiment results and data statistics of NCI-H1688 cells; (**D**) CCK8 assay results of NCI-H1688 cells; (**E**) Protein band diagram and relative protein expression statistics of Ki-67. N=3;**P<0.01

**Figure 7:**
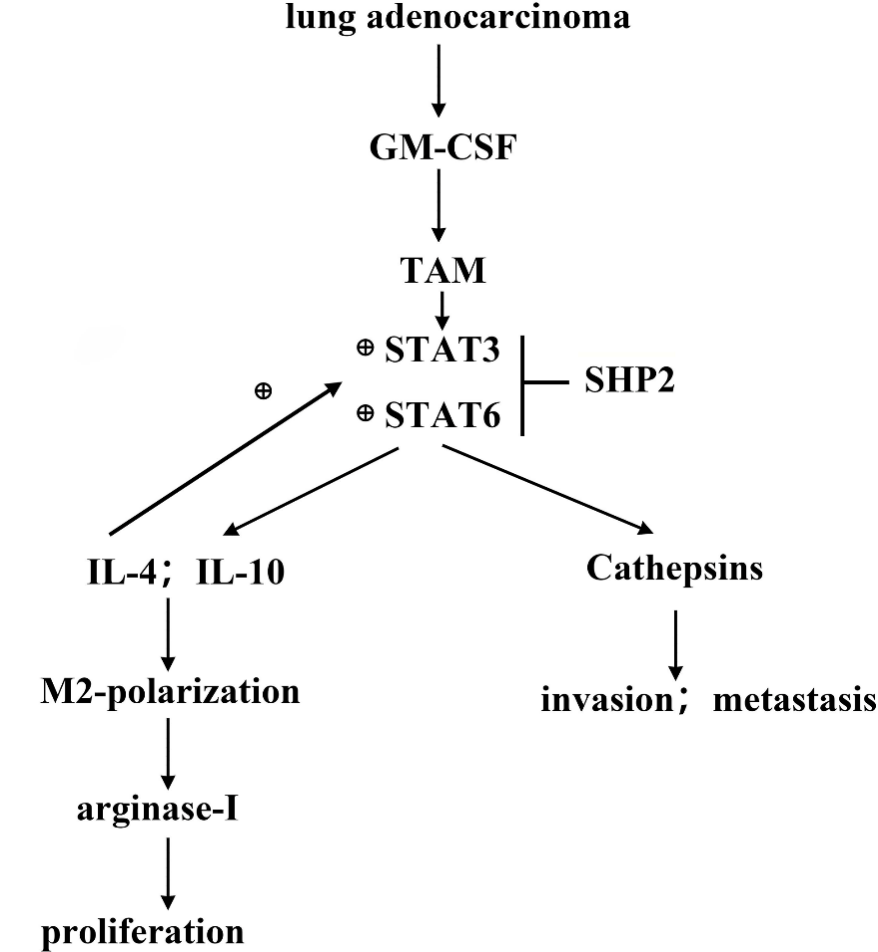
**GM-CSF secreted by lung adenocarcinoma cells can stimulate the activation of STAT3/STAT6 signaling pathway in TAM, and promote the cathepsin secretion and M2 polarization, thus promoting the proliferation and migration of lung adenocarcinoma.** SHP2 can inhibit the development of lung adenocarcinoma by suppressing STAT3.

To evaluate the effect of SHP2 in TAM on the proliferation capacity of lung adenocarcinoma cells, we examine the proliferation capacity of A549 cells and NCI-H1688 cells after coculturing with cell monoclonal assay and CCK8 assay. The results show that the number of monoclonal cells in the sh-RNA SHP2 group is significantly higher than that in the NC group; besides, the OD values in the sh-RNA SHP2 group are significantly higher compared with the NC group in the 48th hour and 72nd hour. The results of Western blotting showed that the relative protein expression of Ki-67 in the sh-RNA SHP2 group was significantly increased in A549 cells and NCI-H1688 cells, compared with the NC group (Figure 6). These results suggest that inhibition of SHP2 in TAM can enhance the proliferation capacity of lung adenocarcinoma cells (Figure 7).

## DISCUSSION

Lung adenocarcinoma, a prevalent form of lung cancer, arises from the glandular cells in lung tissues, representing about 40% of cases. This type often develops in the lung’s outer regions and might not show symptoms early on, leading to late detection. Symptoms can include persistent coughing, coughing up blood, difficulty breathing, chest pain, hoarseness, weight loss, among others [13, 14]. Diagnosis involves a combination of medical history review, physical exams, and imaging tests like X-rays, CT, and PET scans, alongside tissue sample analysis. The disease’s incidence and mortality rates have been climbing, with a significant proportion of patients experiencing metastasis and recurrence post-surgery, affecting survival rates. Treatment strategies encompass surgery, chemotherapy, radiation, targeted therapy, and immunotherapy, with the latter gaining traction for advanced stages [2, 15, 16].

Research on the tumor microenvironment (TME) and its components, such as tumor-associated macrophages (TAM), has been gaining focus. The TME, composed of immune cells, fibroblasts, tumor cells, blood vessels, and the extracellular matrix, is crucial for tumor development and progression. TAMs, significant in the immune cell component, are implicated in tumor progression and are considered therapeutic targets. Their role varies across tumor stages, influencing metastasis, angiogenesis, cell migration, immune cell suppression, and chemoresistance. Macrophages within the TME are polarized into M1 (tumor-suppressive) or M2 (tumor-promoting) phenotypes, highlighting their complex role in cancer dynamics. SHP2, a tyrosine phosphatase, plays a dual role in macrophage function, both promoting and inhibiting anti-tumor activity, thus representing a potential research focus for lung adenocarcinoma treatment strategies. Yet, the relationship between SHP2 and TAM in lung adenocarcinoma remains underexplored, necessitating further research [17–23]. In this study, we investigated the effects of SHP2 inhibition in TAM on lung adenocarcinoma development by coculturing transfected THP1 cells with A549 and NCI-H1688 cells, followed by subcutaneous injection into nude mice. Analyzing TAM sorted via flow cytometry and assessing M2 markers (CD163, CD206, Arginase-1) through Western blot, we observed increased tumor volume and M2 marker expression in the SHP2-inhibited group. These findings suggest that SHP2 inhibition in TAM favors M2 macrophage polarization, thereby potentially accelerating lung adenocarcinoma progression.

Granulocyte-macrophage colony-stimulating factor (GM-CSF), secreted by tumor cells, plays a pivotal role in cancer progression by promoting STAT3 activation in tumor-associated macrophages (TAMs) and inhibiting SHP2 activity [24]. The process of metastasis, a leading cause of mortality in cancer patients, involves the degradation of the extracellular matrix and basement membrane by various proteases, including cathepsins. These enzymes facilitate tumor expansion, angiogenesis, and metastasis [25, 26]. The proliferation marker Ki-67, indicative of cell growth, shows elevated levels in actively dividing tumor cells, contrasting with its low expression in dormant cells.

Experimental results, using techniques such as Western blotting and ELISA, revealed no significant changes in the expression of several markers (p-STAT1, p-STAT3, p-STAT5, p-STAT6, IL-4, IL-10, various cathepsins, and Arginase-1) in the sh-RNA SHP2 group versus control, except for a notable reduction in SHP2 expression. However, in THP-1 cells co-cultured with A549 lung adenocarcinoma cells, the expression of p-STAT3, p-STAT6, and other markers significantly increased, suggesting that GM-CSF from A549 cells can suppress SHP2 in TAMs, thus activating the STAT3/STAT6 pathway and enhancing cathepsin secretion and M2 macrophage polarization.

Further investigations using cell healing and Transwell assays demonstrated that SHP2 inhibition boosts the migratory and invasive capacities of lung adenocarcinoma cells. Monoclonal assays and CCK8 experiments showed that SHP2 suppression leads to a significant increase in the proliferation of A549 and NCI-H1688 cells, corroborated by a marked rise in Ki-67 expression.

In summary, this study illuminates how GM-CSF from lung adenocarcinoma cells modulates the SHP2 function and activates key signaling pathways, contributing to tumor progression by promoting cell proliferation, migration, and macrophage polarization towards an M2 phenotype. These insights could pave the way for novel therapeutic strategies for lung adenocarcinoma treatment.

## MATERIALS AND METHODS

### Cell culture

Human monocytes-macrophages THP1 and human lung adenocarcinoma cells A549 as well as NCI-H1688 are purchased from Procell in Wuhan. THP1 cells are cultured in PRMI-1640 medium with 10% fetal bovine serum and 1% penicillin-streptomycin, and A549 and NCI-H1688 cells are cultured in DMEM medium supplemented with 10% fetal bovine serum and 1% penicillin-streptomycin. All cells are placed in a constant temperature and humidity incubator at 37° C and 5% CO_2_.

### Cell transfection

Lentiviruses encoding shRNA targeting SHP2 as well as negative controls are obtained from GeneChem (Shanghai, China). The lentiviral vector for SHP2 knockdown is induced as well as the corresponding negative control vector. When THP1 cells reach 70% confluence, lentiviral vector transduction is performed using transduction reagent and 8 mg/ml polyvinyl bromide (GeneChem) for 12 hours. THP1 cells are divided into NC group and sh-RNA SHP2 group according to different treatments. After 24-hour incubation, they are subjected to further analysis.

### Cell co-culture

A549 cells and NCI-H1688 cells are inoculated into transwell chambers separately and cultured for 24 hours. THP1 cells are inoculated into cell culture plates and incubated for 24 hours with IXA-4 (40 mg/ml) added to the culture medium. After overnight incubation, the medium in the transwell chambers and culture plates is removed and new culture medium is added to the lower chamber. The transwell chambers are then placed in the cell culture plates and new culture medium is added, and the assay is performed after 24 hours of incubation. The transwell chambers are removed and the culture medium from the cell culture plate is aspirated. The cell culture plates are washed twice with PBS and the cells are fixed with 4% paraformaldehyde.

### Subcutaneous tumor experiment in nude mice

Adult male BALB/c nude mice, 6-8 weeks old, are purchased from the Shanghai Laboratory Animal Research Center and fed at 25±1° C for 12 hours alternating day and night. Besides, adequate food and water are provided. After co-culture, A549 cells with THP1 cell solution and NCI-H1688 cells with THP1 cell solution from each group are injected subcutaneously into the right posterior side of nude mice respectively, and tumor volume and weight are measured and recorded every 7 days. The mice are euthanized at the end of the experiment and tumor tissues are obtained for subsequent experiments.

### ELISA

IL-10 and IL-4 antigens were coated on microplates, 100 μL of antigen was added to each well, left at 37° C for 4 h, and the liquid in the wells was discarded. Then, 5% fetal bovine serum was added and blocked at 37° C for 40 min. After 3 washes, the diluted sample was added to the microwells at a rate of 100 μL per well for 60 minutes. After 3 washes with the washing solution, enzyme-labeled antibodies were added and the reaction was maintained at 37° C for 60 min. After three washes, chromogenic substrate TMB is added to stop the reaction. The OD value was determined with a microplate reader at a wavelength of 450 nm. By plotting a standard curve, the concentration of the sample is calculated.

### Flow cytometry

The tumor tissues of nude mice in each group are isolated under aseptic conditions, washed with D-PBS, and the tissues are cut into 1 mm^3^ size pieces. The tissue pieces are digested with a mixture containing 0.2% type IV collagenase and 0.25% trypsin at 37° C. The supernatant collected after digestion is filtered through a 200-mesh sieve. The cells are resuspended with cell staining buffer (1×PBS containing 1% BSA) and stored at 4° C for future use. The cell density is adjusted to 1×10^6^/100 μl, stained with Rat monoclonal [FA-11] to CD68-APC (Abcam, ab221251) for 30 minutes in the dark. BD FACSAria™ III is used to analyze and sort the cells to remove cell debris and clumps based on cell size. Optimize flow cytometry assay conditions using blank tubes and positive control tubes, and establish sorting gates, gating: forward scatter vs. side scatter (gate 1), live cells (gate 2), CD68 positive cells (gate 3). Data from blank tubes and positive control tubes were collected and compared with the sorted sample data to determine the purity of the sorted samples. Purified expression of positive CD68 macrophages using region markers.

### Cell healing assay

Then the cocultured A549 cells and NCI-H1688 cells are made into cell suspensions and inoculated on six-well plates. 24-hour incubation at 37° C in a 5% CO_2_ incubator is performed. After the cell density reaches 70%, a 100 μL sterile pipette is used to perform the healing experiment with the tip of the scratch as perpendicular to the cells as possible to ensure that the width of the scratch is essentially the same for each group. The cells are washed twice with PBS and then incubated for 24 hours at 37° C in 5% CO_2_ serum-free medium. Photographs are taken at 0h and 48h respectively to measure the healing distance.

### Transwell

Migration experiment: A549 cells and NCI-H1688 cells are resuspended in serum-free medium. 100 μL of cell suspension is added to the upper chamber of Transwell, and 600 μL of serum-containing medium is added to the lower chamber for 48 hours. The cells on the top surface of the membrane that have not migrated are removed with cotton swabs, and the cells on the lower surface of the membrane are fixed with paraformaldehyde. After washing, they are stained with crystal violet. After washing and drying, the cells are counted under an inverted microscope. Invision experiment: Matrigel is diluted with serum-free medium at a ratio of 1:8, wrapped around the upper surface of the membrane at the bottom of the Transwell, and incubated in an incubator at 37° C for 4 hours to polymerize Matrigel into gels for later use. The rest of the procedure is the same as the Migration experiment.

### CCK8

A549 cells and NCI-H1688 cells in logarithmic growth phase are digested and diluted, and then the cells are counted with a cell counting plate. Cell suspension with density of 5×10^4^/ml is prepared with complete culture medium, and 100 ul/well is inoculated into a 96-well plate and placed in an incubator at 37° C with 5% CO_2_. After the cells are plastered, they are incubated for 24h, 48h and 72h respectively. 100 ul of fresh complete medium is used to replace the old medium, and 10 ul of CCK-8 reagent is added to each well. The plates are incubated in the dark for 2 hours, and cell viability is detected at 450 nm on a microplate reader. The experiment is repeated 3 times. Its statistical analysis is performed and plotted.

### Monoclonal proliferation

A549 cells and NCI-H1688 cells in good growth condition are taken. When the cells reach 80% confluence in the 6-well plate, the cells are routinely digested and centrifuged at 1200 rpm for 5 minutes. Resuspend the cell pellet, count the cells, adjust the cell concentration to 1.0×10^5^, and set 3 replicate culture dishes for each group. Take 200 μL of cell suspension to inoculate the petri dishes, and replenish the cell culture medium to 6 mL. Shake in a cross shape to disperse the cells evenly, put them into 37° C, 5% CO_2_ incubator for 2 weeks of conventional incubation, and discard the medium. Wash 3 times with PBS. Add 5mL of methanol to each well for fixation for 15 minutes, and discard the fixative. After adding Giemsa solution to each well for 20 minutes, rinse with tap water and dry in the air. Take pictures of each well, file, and use Image J software for cell counting.

### Co-immunoprecipitation

Follow the manufacturer’s guidelines (Takara Biotechnology, Dalian, China) using the Pierce™ Direct IP Kit 26148. Add 20 μL of Amino Link PlusResin and Pierce Control Agarose Resin (a negative control to eliminate non-specific protein binding) with 4 μg of anti-SHP2 rabbit monoclonal antibody for 100 minutes. Subsequently, a protein sample (500 μL) was added and incubated overnight, followed by four rinses with IP lysis/wash buffer and once with pretreatment buffer. The SHP2 protein complex was then eluted with solution buffer, 10% SDS-PAGE electrophoresis (110 V, 25 min electrophoresis time), and then Western blotting as described above. The membranes were incubated with SHP2 (1:1,000), p-STAT3 (1:1,000), and p-STAT6 (1:1,000) primary antibodies overnight at 4° C. After three washes in TBST, the membrane is developed using an enhanced chemiluminescent (ECL) reagent.

### Western blot

After removing the culture medium, 500 μl protein lysate is added to each well for lysis for 30 minutes, centrifuged at 4° C, 1500 r/min for 5 minutes. And the supernatant is taken to detect the protein concentration of the sample. Transfer the protein to PVDF membrane through Gels preparation, samples loading, electrophoresis and wet transfer and close the blocking solution for 1 hour. Sequentially, primary antibody SHP2 (Abcam, ab187040, 1:5000), Ki-67 (Abcam, ab231172, 1:5000), p-STAT1 (Abcam, ab109461, 1:1000), p-STAT3 (Abcam, ab76315, 1:1000), p-STAT5 (Abcam, ab278764, 1:1000), p-STAT6 (Abcam, ab263947, 1:1000), IL-4 (Abcam, ab34277, 1:1000), IL-10 (Abcam, ab52909, 1:1000), Cathepsin-L (Abcam, ab200738, 1:1000), Cathepsin-S (Abcam, ab134157, 1:1000), Cathepsin-K (Abcam, ab207086, 1:1000), Arginase-1 (Abcam, ab133543, 1:1000) and GAPDH (Abcam, ab181603, 1:10000) are added and the secondary antibody is incubated for 2 hours at room temperature. Exposure imaging is performed and Quantity One V4 software is used for analysis.

### Statistical analysis

Statistical analysis of the data is performed using SPSS22.0 software. Graphs are made with Graphpad prism Software 9.0 software, and the results are expressed as (mean±SEM). t-test is used to compare the two groups. The experiment is repeated at least three times for each group, and P < 0.05 is regarded as statistically significant.

### Availability of data and materials

Additional data and materials may be requested from the corresponding author on reasonable request.
